# The Differential Gene Expression Pattern of *Mycobacterium tuberculosis* in Response to Capreomycin and PA-824 versus First-Line TB Drugs Reveals Stress- and PE/PPE-Related Drug Targets

**DOI:** 10.1155/2009/879621

**Published:** 2009-07-22

**Authors:** Li M. Fu, Shu C. Tai

**Affiliations:** Pacific Tuberculosis and Cancer Research Organization, P. O. Box 9706, Anaheim, CA 92812, USA

## Abstract

Tuberculosis is a leading infectious disease causing millions of deaths each year. How to eradicate mycobacterial persistence has become a central research focus for developing next-generation TB drugs. Yet, the knowledge in this area is fundamentally limited and only a few drugs, notably capreomycin and PA-824, have been shown to be active against non-replicating persistent TB bacilli. In this study, we performed a new bioinformatics analysis on microarray-based gene expression data obtained from the public domain to explore genes that were differentially induced by drugs between the group of capreomycin and PA-824 and the group of mainly the first-line TB drugs. Our study has identified 42 genes specifically induced by capreomycin and PA-824. Many of these genes are related to stress responses. In terms of the distribution of identified genes in a specific category relative to the whole genome, only the categories of PE/PPE and conserved hypotheticals have statistical significance. Six among the 42 genes identified in this study are on the list of the top 100 persistence targets selected by the TB Structural Genomics Consortium. Further biological elucidation of their roles in mycobacterial persistence is warranted.

## 1. Introduction

Tuberculosis (TB) is a deadly infectious disease caused by *Mycobacterium tuberculosis* that typically affects the lungs (pulmonary TB) but may also occur in other organs (extrapulmonary TB), such as the central nervous system, lymphatic system, circulatory system, genitourinary system, bones, joints, and the skin. *M. tuberculosis* infection is extremely difficult to treat mainly because of its adaptive ability to turn a hostile environment within human macrophages (phagocytes) into a friendly niche for its replication. The bacilli can persist in human tissues, at the primary or secondary infection sites, for a long period of time without multiplication and later be reactivated when the host immune system is compromised. Persistent bacilli refer to those nongrowing bacilli that, often derived from in vivo, can grow immediately in a fresh medium [1]. Bacterial persistence in vivo is analogous to the stationary phase culture in vitro [2]. Nonreplicating persistence of tubercle bacilli can be induced under hypoxia [3]. Dormant bacilli refer to nongrowing bacilli that do not grow immediately in a fresh medium but can be resuscitated [1]. Latent TB infection (LTBI) is a clinical condition associated with only a positive tuberculin skin test (i.e., evidence of infection with *M. tuberculosis*) but without clinical or radiographic signs of active disease. Persons with LTBI are at increased risk for development of active disease, which may occur after decades of latent infection [4]. From the above definitions, the distinction between persistent and dormant bacilli seems to be their rates of recovery from the nongrowing state. A recent study shows that *M. tuberculosis* replicates throughout the course of chronic TB and is restrained by the host immune system [5], which finding suggests that the switch between the nonreplicating and slow replicating states is a dynamic process subject to host immunity. In the text, we do not make distinction between “nonreplicating persistence” and “dormancy”. The presence of dormant tubercle bacilli in animal tissues is best demonstrated in a well-known Cornell model [6]. In this model, some mice infected with *M. tuberculosis* and then treated with a short-term chemotherapy (INH plus PZA) for 3 months developed a relapse after discontinuation of the therapy. Dormancy can cause phenotypic drug resistance. In the Cornell model [6], the bacilli recovered from the relapsed mice were fully susceptible to INH and PZA, indicating phenotypic rather than genetic drug resistance associated with dormant bacilli.

 Eradicating mycobacterial persistence would be an indispensable element for development of next-generation TB drugs. Recently, much research effort has been focused on testing capreomycin and PA-824 for TB treatment with emphasis on their unique bactericidal effect on persistent tubercle bacilli. Capreomycin is an old antibiotic for treatment of pulmonary tuberculosis [7] with recently increasing interest. Capreomycin is effective against (Multi-Drug resistant) MDR and intracellular TB bacilli [8]. A recent study demonstrates that among known anti-TB drugs, only capreomycin is active against nonreplicating *M. tuberculosis* bacilli in an in vitro model of persistence [9]. Capreomycin is thus important because it can deal with both MDR and latent TB. PA-824 exhibits bactericidal activity against both replicating and static (persistent) *tuberculosis* and it also has potent bactericidal activity against MDR *M. tuberculosis* in animal infection models [10]. PA-824 shows a better sterilizing activity than moxifloxacin [11] and could enhance bactericidal activity of rifampin and/or pyrazinamide [12]. Both capreomycin and PA-824 inhibit protein synthesis [10, 13]; PA-824 also inhibits the synthesis of cell wall lipid [10]. Yet, how these two drugs eradicate persistent bacilli is unclear.

 In this work, we conducted an exploratory analysis of differential gene expression in response to capreomycin and PA-824 versus the current first-line TB drugs in an attempt to identify drug targets potentially linked to the unique drug action against nonreplicating TB bacilli. Our study found that the genes significantly differentially expressed in response to capreomycin and PA-824 were dominated by PE/PPE or conserved hypotheticals, and many of these genes were related to stress responses. These genes and their products may serve as new drug targets for TB drug development.

## 2. Materials and Methods

We performed differential gene expression analysis between two sets of data samples such that one set of samples was derived from drugs that were bactericidal to nonreplicating persistent TB bacilli (defined as the goal property) and the other set of samples from drugs that lacked this property. The first set of samples served as the experimental group whereas the second set as the control group. Only a few drugs have experimentally demonstrated the bactericidal effect on persistent *M. tuberculosis* grown in an anaerobic or hypoxic condition. In the present study, the experimental group consisted of two best-known drugs in this category: capreomycin and PA-824. All first-line TB drugs were selected to be included in the control group except (pyrazinamide) PZA for the reason stated later. A second-line TB drug, ethionamide, and a non-TB drug, ampicillin, were also added to the control group. Fluoroquinolones were not included in this study because current evidence is inconsistent about their activity against persistent TB bacilli.

## 3. Data Collection

The microarray-based gene expression data were collected from the GEO (Gene Expression Omnibus) database at NCBI (National Center for Biotechnology Information) of NIH (National Institutes of Health) at http://www.ncbi.nlm.nih.gov/geo/ In GEO, the data are stored in a relational database with three top-level entity types: platforms, samples, and series. A platform describes the format or model of the microarray (e.g., oligonucleotides, cDNAs, etc.). A sample describes the gene expression data of a single hybridization under a given experimental condition. A series consists of the related samples in an experiment. The original data were generated by treating *M. tuberculosis * H37Rv with a wide variety of metabolic inhibitors, including TB drugs, using a cDNA platform in a study conducted by Boshoff et al. [14], where the details of original data collection can be found. The gene expression data of the present study comprised samples of the following accession numbers: GSM28106, GSM28096, GSM28246, GSM28245, GSM28062, GSM28055, GSM28037, GSM28060, GSM28065 and GSM28063, which were collected under the series GSE1642.

## 4. Significance Analysis of Microarrays

The (Significance Analysis of Microarrays) SAM program can effectively recognize differentially expressed genes with high statistical significance from multiple data sets of two or more classes by using balanced perturbation of repeated measurements and minimization of the false discovery rate (FDR) [15]. FDR, as an alternative to the traditional *P*-value, has been widely accepted for gene selection from microarray data. Through balanced perturbation of repeated measurements, SAM proves to be more robust than traditional statistical tests such as the *t-*test. In the present study, we applied SAM version 3.0 to determine significant genes that were differentially expressed in gene expression profiling between the experimental and control groups. The data used in the present study, organized in the excel format ready to be processed by SAM, can be downloaded from our web site (http://www.patcar.org/Research/IJMB-2009.html). The data were processed by SAM with the following parameter settings: two-class, unpaired, log-ratio, and FDR set to 0.05.

## 5. Significance Analysis of Genes in Functional Categories

The chance probability of identifying *n* genes among which *i* genes belong to a functional category of size *f* is computed by the following adapted formula [16]:

(1) $$P=\frac{\left(\begin{array}{c} f\\ i \end{array}\right)\left(\begin{array}{c} g-f\\ n-i \end{array}\right)}{\left(\begin{array}{c} g\\ n \end{array}\right)},$$

where *g* is the total number of genes in the genome.

## 6. Results

### 6.1. Genes Differentially Expressed in Capreomycin and PA-824

SAM analysis on two classes of gene expression samples yielded positive and negative significant genes. Positive significant genes refer to genes more strongly induced in the experimental group than in the control group, whereas negative significant genes are genes less strongly induced in the experimental group compared with the control group. In this study, the four gene-expression data samples concerning the responses of *M. tuberculosis * to capreomycin and PA-824 at a low and high doses constituted the experimental group, and the rest of data samples representing the responses of *M. tuberculosis * to other selected drugs is assigned to the control group. Consequently, positive significant genes are genes that are differentially expressed in response to capreomycin and PA-824. Since capreomycin and PA-824 are bactericidal to nonreplicating TB bacilli, genes induced by these two drugs but not by the other drugs in the control group would likely be linked to bactericidal activity against nonreplicating TB bacilli, as this is the main attribute that differentiates between the two groups.

**Figure 1:**
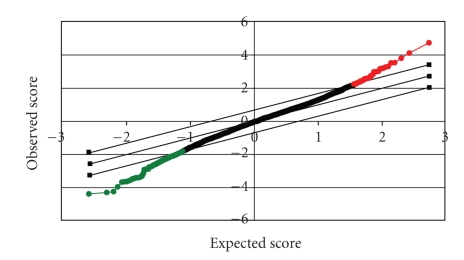
The SAM plot where the significant genes are distributed at the two ends. Significant: 242, Median number of false positives: 11.31, False Discovery Rate (%): 4.67, Tail strength (%): 36.2, and se (%): 25.1.

 SAM analysis on the gene expression data collected for this study generated 42 positive significant genets and 196 negative significant genes (Figure 1). Genes were removed from consideration if they were not induced by capreomycin or PA-824. In the drug-treated gene-expression profiling experiment, induced genes are more significant than repressed genes since the induction of certain genes reflects the feedback or compensatory mechanism that senses the interruption of the drug-acting metabolic pathways in which the genes are involved [17]. Our prior data also suggest that whereas the molecular characteristics of the induced genes reflect the drug's mode of action, the repressed genes are often nonspecific or secondary [18]. Therefore, we focused our attention on the positive genes. Those genes differentially induced by capreomycin and PA-824 are displayed in Table 1 and their gene expression values across all the drugs are summarized in Table 2. Functional analysis of these genes would provide further insight into the main issue.

### 6.2. Functional Analysis of Identified Genes

The genes differentially expressed in response to capreomycin and PA-824 fall in seven functional categories (Table 3). The number of the genes identified from a functional category is considered statistically significant at a level of significance of 0.05 if the associated chance probability is less than 0.005 after the adjustment for the multiplicity effect due to 10 categories. By this criterion, PE/PPE and conserved hypotheticals are the only categories with statistical significance, while identification of the genes in other categories could be explained by chance.

 The persistence-related genes identified in this study were interpreted in terms of their functions and roles based on current research evidence (Table 4). These genes are involved in stress (hypoxia, starvation, or heat-shock) responses, fatty acid degradation/catabolism for energy utilization, membrane function, survival (i.e., essential genes), growth, or sulfur metabolism. Yet, many genes identified in the present study are conserved hypotheticals with unknown function. Further biological validation on these genes is warranted.

## 7. Discussion

In the present study, we collected two sets of gene expression data samples from the GEO database so that one set of samples was derived from drugs that were active against nonreplicating persistent TB bacilli and the other set of samples from drugs that were not. We used a standard differential gene expression analytical method [25] developed in the field of functional genomics [26]. The gene expression data were generated in a study that was aimed to gain new insight into drug mechanisms of action by performing a large scale of gene expression profiling experiments concerning *M. tuberculosis* responses to a variety of metabolic inhibitors [14]. Their study, however, did not address the mechanisms against nonreplicating *M. tuberculosis * as we did in the present study.

 PZA is used in combination with other first-line TB drugs in order to reduce the required treatment duration [27]. In addition, PZA in conjunction with rifampin can be used to treat latent tuberculosis [28]. These two facts suggest that a potential role PZA may play in killing persistent TB bacilli, despite that PZA alone did not show this property experimentally [9]. Because of this uncertainty, PZA was not included in the control group.

 The data were generated from gene expression experiments carried out on aerobic growing bacilli rather than on anaerobic dormant bacilli. Research based on global gene expression profiling has identified genes that are differentially expressed by *M. tuberculosis* resident in macrophages compared with *M. tuberculosis * grown in standard broth culture [29]. Despite recent progress in this area, the genetic responses of intracellular *M. tuberculosis* exposed to drugs have not yet been studied. Thus, the present study was limited by this fact. Research shows that gene expression in a hypoxic condition reflects the fact that many metabolic pathways are repressed and specific hypoxic response pathways are turned on. In our prior study on capreomycin, the upregulation of isocitrate lyase (ICL) suggests that capreomycin can affect the glyoxylate shunt, which is a pathway alternative to the tricarboxylic acid (Krebs) cycle and is involved in intracellular mycobacterial persistence when fatty acids become a major source of carbon and energy in *M. tuberculosis* metabolism [30]. Although that study was conducted in an aerobic condition, the gene expression pattern found there also revealed responses to the nonreplicating state due to the drug's action.

 Another limiting factor in the present study is due to the fact that the observed bactericidal effect of capreomycin and PA-824 on nonreplicating bacilli is based on the Wayne model [3], which has been widely adopted for this purpose, but its accuracy for simulating the in vivo nonreplicating state has been questioned. Despite the above limitations, the present findings would serve as a basis for future work.

 The pathogenicity of *M. tuberculosis* involves a complex set of factors. The availability of the *M. tuberculosis* genome sequence coupled with advances in molecular biology has resulted in a wide range of novel drug targets related to transcription, cell wall synthesis, signal transduction, information pathway, intermediate metabolism, virulence, and persistence [31]. The need of lengthy chemotherapy reflects the lack of adequate understanding in bacterial persistence. However, a finding that the persistence of *M. tuberculosis* in mice requires the glyoxylate shunt enzyme isocitrate lyase (ICL) [32] points to a new drug target. Other possible drug targets related to TB persistence are DosR, RelA, and PcaA [31]. As there have been only a handful of drug targets hypothesized as relevant to mycobacterial persistence to date and the prospect of their serving as a basis for developing a practically useful new TB drug is uncertain, our study is aimed to address this issue by taking a genomewide approach to finding new TB drug targets.

 The in vivo microenvironment of persistent TB bacilli is often located in such lesions as granulomas, cavities, or caseous tissue necrosis, where oxygen and nutrients are both deprived. Stress results from hypoxia and starvation as well as host immunity. To survive, TB bacilli must activate certain metabolic pathways to deal with the stress. Such rescue pathways have been studied for hypoxia [33], starvation [20], and high temperature [21].

 Six among the 42 genes identified in the present study are on the list of the top 100 persistence targets selected by the TB Structural Genomics Consortium (http://www.webtb.org/). These genes are: Rv2557, Rv1285, Rv2878C, Rv2777C, Rv1929C, and Rv0834C. The chance probability of identifying 42 genes from all genes in the *M. tuberculosis* genome such that 6 genes are in the category of the 100 persistence-related genes is.0005, and hence it is highly statistically significant in this sense. It is not clear how the relevance to persistence is assessed for those top 100 persistence targets, but it is presumably based on evidence derived from long-term research efforts on *M. tuberculosis * conducted by the Consortium members. There is no guarantee that these top targets are the best drug targets for future TB drug development. Nevertheless, the overlap of our findings with the data provided by the Consortium is significant and offers credence to the findings.

 It is interesting to note that none of the identified genes belong to information pathways or to regulatory proteins. Some well-known TB drugs target information pathways. For example, rifampin targets RNA polymerase, fluoroquinolones target DNA gyrase, and streptomycin targets ribosomal protein and 16S rRNA. These drugs are effective against actively growing TB bacilli, but they cannot eradicate persistent bacilli. In contrast, capreomycin and PA-824 target not only information pathways but also other metabolic pathways. Our analysis suggests that inhibition of the information pathway alone is not sufficient for resolving the issue of mycobacterial persistence.

 The distribution of the genes identified in the categories of virulence, lipid metabolism, cell wall and cell processes, intermediary metabolism and respiration is not statistically significantly different from their distribution in the whole genome. Thus, the significance of these gene categories as relevance to mycobacterial persistence is questionable. For example, isoniazid, the best known first-line TB drug, works by inhibiting mycobacterial cell wall synthesis but it is not effective against persistent TB bacilli [9].

 PE/PPE and conserved hypotheticals are the only two functional categories where the distribution of the genes identified in this study is significantly different from their distribution in the whole genome. This finding suggests that a significant number of genes pertinent to persistence elimination are likely PE/PPE or conserved hypothetical proteins. If this hypothesis turns out to be true, then it explains why the persistence problem is difficult to solve since the current knowledge about genes in these two categories is quite limited. Elucidation of the functions of these PE/PPE and hypothetical proteins could result in information useful for developing new TB drugs. The investigation of some of these genes related to persistence is currently underway, for example, Rv2557, Rv2777c, and Rv1929c in the hypothetical category, and Rv0834c in the PE family.

 About 10% of the genes in *M. tuberculosis * genome are dedicated to the production of two families of glycine-rich proteins called PE (proline-glutamine motifs) and (proline-proline-glutamine motifs) PPE, characterized by repetitive structure that may represent a source of antigenic variation [24]. It is logical to assume that suppression of the PE/PPE synthesis pathway may reduce antigenic variation and thereby facilitate the bactericidal mechanisms due to host immunity; however, this hypothesis remains to be proven.

## 8. Conclusion

The treatment of *M. tuberculosis* infection is often difficult mainly because of its adaptive ability to persist in the host tissue for a long period of time. Eradicating mycobacterial persistence would be an indispensable element for developing next-generation TB drugs. Yet, our understanding of the mechanisms of persistence is still far from reaching the point where an effective new drug can be developed to solve the problem. In fact, only a handful of genes are found to be potential drug targets pertinent to mycobacterial persistence, and only a few drugs exhibit bactericidal activity against nonreplicating TB bacilli, notably, capreomycin and PA-824. In the present study, we identified genes differentially expressed in response to capreomycin and PA-824 versus the first-line TB drugs. Six genes identified in our study are on the list of the top 100 persistence targets selected by the TB Structural Genomics Consortium. A significant number of genes identified are PE/PPE or conserved hypotheticals, and many of these genes are related to stress responses. Further biological validation on these genes is warranted.

## Figures and Tables

**Table 1:** Genes differentially induced in response to capreomycin and PA-824 relative to other drugs.

ORF	Gene	Product/Function	Score	*q*-value %
Rv2657c	—	Probable phirv2 prophage protein	4.73	0
Rv2374c	*hrcA*	Probable heat shock protein transcriptional repressor	4.12	0
Rv0354c	PPE7	PPE family protein	3.80	0
Rv1364c	—	Conserved hypothetical protein	3.54	0
Rv2304c	—	Hypothetical protein	3.52	0
Rv0367c	—	Hypothetical protein	3.30	0
Rv0607	—	Hypothetical protein	3.26	0
Rv2466c	—	Conserved hypothetical protein	3.21	0
Rv0830	—	Conserved hypothetical protein	3.18	0
Rv2557	—	Conserved hypothetical protein	3.16	0
Rv2142c	—	Hypothetical protein	3.02	0
Rv0654	—	Probable dioxygenase	2.99	0
Rv1977	—	Conserved hypothetical protein	2.99	0
Rv2688c	—	Probable antibiotic-transport ATP-binding protein	2.98	0
Rv1285	*cysD*	Probable sulfate adenylyltransferase subunit 2	2.82	1.04
Rv0047c	—	Conserved hypothetical protein	2.77	1.73
Rv2878c	*mpt*53	Soluble secreted antigen mpt53 precursor	2.74	1.73
Rv2254c	—	Probable integral membrane protein	2.63	1.86
Rv1986	—	Probable conserved integral membrane protein	2.60	1.86
Rv2777c	—	Conserved hypothetical protein	2.60	1.86
Rv2413c	—	Conserved hypothetical protein	2.59	1.86
Rv1929c	—	Conserved hypothetical protein	2.59	1.86
Rv3288c	*usfY*	Putative protein	2.57	1.86
Rv2535c	PEPQ	Probable cytoplasmic peptidase	2.54	2.27
Rv2634c	PE_PGRS46	PE_PGRS family protein	2.54	2.27
Rv1646	PE17	PE family protein	2.49	2.72
Rv2749	—	Conserved hypothetical protein	2.46	2.72
Rv3452	*cut*4	Probable cutinase precursor cut4	2.44	3.22
Rv0192	—	Conserved hypothetical protein	2.41	3.22
Rv1687c	—	Probable conserved ATP-binding protein ABC transporter	2.41	3.22
Rv1087	PE_PGRS21	PE_PGRS family protein	2.37	4.35
Rv1566c	—	Possible inv protein	2.36	4.35
Rv2504c	*scoA*	Probable succinyl-coa3-ketoacid-coenzyme A transferase	2.35	4.35
Rv0834c	PE_PGRS14	PE_PGRS family protein	2.33	4.35
Rv0274	—	Conserved hypothetical protein	2.33	4.35
Rv2162c	PE_PGRS38	PE_PGRS family protein	2.30	4.35
Rv0365c	—	Conserved hypothetical protein	2.30	4.35
Rv3550	*echA*20	Probable enoyl-CoA hydratase	2.27	4.67
Rv3367	PE_PGRS51	PE_PGRS family protein	2.26	4.67
Rv2802c	—	Hypothetical arginine and alanine rich protein	2.25	4.67
Rv2291	*sseB*	Probable thiosulfate sulfurtransferase	2.25	4.67
Rv3189	—	Conserved hypothetical protein	2.25	4.67

*Score: the *t*-statistic score. *q*-value: the level of significance based on SAM.

**Table 2:** The expression values (log_2_ ratios) of genes more strongly induced by capreomycin and PA-824 than the other drugs.

ORF	Gene	Exp1	Exp2	Exp3	Exp4	Cont1	Cont2	Cont3	Cont4	Cont5	Cont6
Rv2657c	—	1.614	1.825	2.209	1.278	−0.3	−0.81	−0.54	0.42	−0.29	−0.67
Rv2374c	*hrcA*	1.472	1.747	1.461	0.375	−0.36	−0.58	−0.57	−0.31	−0.41	−0.56
Rv0354c	PPE7	0.855	0.881	0.777	1.236	−0.51	0	−0.63	0	−0.58	−0.37
Rv1364c	—	1.309	1.01	0.685	1.05	−0.03	0.081	0.069	−0.42	−0.52	−0.36
Rv2304c	—	0.893	1.17	1.051	1.16	0.115	−0.29	−0.31	0.35	0.015	−0.01
Rv0367c	—	1.018	1.198	1.933	1.452	0.057	−0.02	0.093	0.647	0.103	−0.29
Rv0607	—	0.978	0.362	0.828	0*	−0.77	−0.39	−0.78	−0.69	−0.77	−1.33
Rv2466c	—	2.283	3.242	0.831	1.869	−0.06	0.1	0.025	0.755	−0.01	0.022
Rv0830	—	1.305	0.786	0.748	0.78	−0.13	0.137	−0.08	0.041	−0.26	0*
Rv2557	—	0.417	1.064	1.104	1.28	−0.07	0.039	0.064	−0.34	−0.15	−0.25
Rv2142c	—	2.194	2.353	0.92	1.099	−0.35	00*	−0.18	−1.24	0.668	00*
Rv0654	—	2.3	1.345	1.277	0.908	−0.13	−0.95	−0.53	0.726	−0.04	−0.08
Rv1977	—	0.395	0.434	0.537	0.61	−0.28	−0.65	−0.23	−0.36	−0.1	−0.32
Rv2688c	—	1.033	1.686	1.289	2.103	0.329	−0.15	0.063	0.73	0.093	0.547
Rv1285	*cysD*	2.109	1.628	2.463	0.746	−0.2	0.158	−0.15	1.181	−0.13	−0.16
Rv0047c	—	1.31	1.796	1.446	1.461	−0.09	−0.29	1.12	−0.52	−0.29	0.633
Rv2878c	*mpt*53	0.639	1.564	0.473	0.733	−0.27	−0.21	−0.68	0.013	−0.4	−0.01
Rv2254c	—	1.795	2.008	0.324	0.305	−0.2	−0.23	−0.76	−0.62	−0.07	−0.34
Rv1986	—	2.467	2.354	1.131	0.666	0.295	0*	0.096	0.814	−0.15	0*
Rv2777c	—	−0.26	2.052	1.453	0.792	−0.88	−0.11	−0.74	−0.21	−1.17	−0.46
Rv2413c	—	0.854	0.565	1.435	0.438	0.012	−0.06	−0.71	−0.09	−0.03	−0.4
Rv1929c	—	0.71	0.581	1.359	0.721	0.004	−0.12	0.078	0.172	0.023	−0.31
Rv3288c	*usfY*	1.688	1.397	1.253	0.31	0.153	0.325	−0.09	0.191	−0.12	−0.07
Rv2535c	PEPQ	0.646	0.956	−0.07	0.451	−0.53	−0.2	−0.16	−0.61	−0.77	−0.63
Rv2634c	PE_PGRS46	0.673	0.494	0.473	0.765	−0.09	−0.39	−0.03	0.028	−0.03	−0.56
Rv1646	PE17	1.947	2.987	0.451	1.528	−0.24	0*	−0.32	1.113	−0.48	0.124
Rv2749	—	0.669	0.611	0.597	1.069	0.138	0.11	0.083	0.014	0.065	0.118
Rv3452	*cut*4	0.753	0.932	1.36	0.673	0.244	0.259	0.097	0.176	0.156	0.271
Rv0192	—	0.74	1.674	1.227	0.694	0.424	0.158	−0.04	0.044	0.365	0.077
Rv1687c	—	0.894	0.446	1.547	0.726	0.19	0.177	0.051	−0.33	−0.02	−0.33
Rv1087	PE_PGRS21	0.553	0.361	0.797	0.474	−0.1	0.075	−0.14	−0.24	0.039	−0.28
Rv1566c	—	1.732	1.274	1.451	1.097	0.513	−0.16	0.835	−0.61	0.685	0.352
Rv2504c	*scoA*	0.675	0.915	1.978	0.376	−0.28	0.095	−0.66	0.396	−0.49	−0.38
Rv0834c	PE_PGRS14	0.69	1.236	0.827	1.372	0.147	0.417	0.382	0.455	0.141	0.073
Rv0274	—	0.189	0.424	1.123	1.799	−0.19	0.002	−0.49	−1.47	−0.11	−0.41
Rv2162c	PE_PGRS38	0.631	1.093	0.436	0.478	−0.1	−0.14	−0.34	0.191	−0.04	−0.04
Rv0365c	—	0.134	0.174	0.808	0.243	−0.43	−0.15	−0.64	−0.5	−0.24	−0.57
Rv3550	ECHA20	1.082	−0.22	1.7	1.633	−0.33	0.121	−0.5	−0.09	−0.35	0*
Rv3367	PE_PGRS51	0.065	0.223	0.874	0.57	−0.57	0.197	−0.49	−0.53	−0.63	−0.71
Rv2802c	—	−0.15	0.542	0.319	0.016	−0.31	−0.5	−0.95	−0.42	−0.66	−0.68
Rv2291	*sseB*	0.528	0.31	1.646	0.867	−0.18	−0.09	−0.35	−0.01	−0.04	0.071
Rv3189	—	2.111	1.681	3.122	1.608	−0.43	0.287	−0.22	1.945	1.349	0*

Exp1-2: capreomycin. Exp3-4: PA-824. Cont1: INH. Cont2: rifampin. Cont3: ethambutol. Cont4: streptomycin. Cont5: ethionamide. Cont6: Ampicillin.

*The default value in the case of missing values.

**Table 3:** Functional classes of genes strongly induced by capreomycin and PA-824 relative to other TB drugs. *P* = the chance probability of the number of the genes identified from a given functional category among all genes. Functional classes are based on TubercuList (http://genolist.pasteur.fr/TubercuList/).

Functional class	Induced genes	Induced gene No. (*P* *-*value)	All genes in the Class
0 virulence, detoxification, adaptation	Rv2374C Rv1566C	2 (0.275)	212
1 lipid metabolism	Rv2504C Rv3550	2 (0.264)	237
2 information pathways	None	0 (N/A)	232
3 cell wall and cell processes	Rv2688C Rv2878C Rv2254C	6 (0.129)	751
	Rv1986 Rv3452 Rv1687C		
4 stable RNAs	None	0 (N/A)	50
5 insertion seqs and phages	Rv2657C	1 (0.333)	147
6 PE/PPE	Rv0354C Rv2634C Rv1646	7 (0.001)	168
	Rv1087 Rv0834C Rv2162C		
	Rv3367		
7 intermediary metabolism & respiration	Rv0654 Rv1285 Rv2535C	4 (0.018)	898
	Rv2291		
8 unknown	None	0 (N/A)	15
9 regulatory proteins	None	0 (N/A)	194
10 and 16 conserved hypotheticals	Rv1364C Rv2466C Rv0830	20 (0.0045)	1157
	Rv1977 Rv0047C Rv2777C		
	Rv2413C Rv1929C Rv2749		
	Rv0192 Rv0274 Rv0365C		
	Rv3189 Rv2304C Rv0367C		
	Rv0607 Rv2557 Rv2142C		
	Rv3288C Rv2802C		

**Table 4:** Current evidence linking genes identified in this study to * M. tuberculosis* persistence.

**ORF**	**Gene**	**Existing Evidence**
Rv2657c	—	Unknown
Rv2374c	*hrcA*	Required for survival in primary murine macrophage [19]
		Regulation of heat-shock proteins
Rv0354c	PPE7	Downregulated after 96 hours of starvation [20]
Rv1364c	—	Unknown
Rv2304c	—	Unknown
Rv0367c	—	Upregulated at high temperatures [21]
Rv0607	—	Essential gene [22]
Rv2466c	—	Upregulated at high temperatures [21]
Rv0830	—	Unknown
Rv2557	—	Upregulated after 24 hours and 96 hours of starvation [20]
		Expression in human lung granulomas [23]
Rv2142c	—	Unknown
Rv0654	—	Upregulated at high temperatures [21]
Rv1977	—	Unknown
Rv2688c	—	Unknown
Rv1285	*cysD*	Upregulated at high temperatures [21]
		Upregulated after 24 hours of starvation [20]
Rv0047c	—	Unknown
Rv2878c	*mpt*53	Unknown
Rv2254c	—	Unknown
Rv1986	—	Unknown
Rv2777c	—	Upregulated at high temperatures [21]
Rv2413c	—	Upregulated after 4 hours of starvation [20]
Rv1929c	—	Upregulated after 96 hours of starvation [20]
Rv3288c	*usfY*	Upregulated after 24 hours and 96 hours of starvation [20]
Rv2535c	PEPQ	Impaired growth [22]
Rv2634c	PE_PGRS46	Unknown
Rv1646	PE17	Unknown
Rv2749	—	Unknown
Rv3452	*cut*4	Unknown
Rv0192	—	Unknown
Rv1687c	—	Unknown
Rv1087	PE_PGRS21	Unknown
Rv1566c	—	Unknown
Rv2504c	*scoA*	Involved in fatty acid degradation and synthesis [24]
Rv0834c	PE_PGRS14	Unknown
Rv0274	—	Upregulated after 4 hours, 24 hours and 96 hours of starvation [20]
Rv2162c	PE_PGRS38	Unknown
Rv0365c	—	Involved in survival in macrophages [19]
Rv3550	ECHA20	Fatty acid degradation [24]
Rv3367	PE_PGRS51	Upregulated after 96 hours of starvation [20]
Rv2802c	—	Unknown
Rv2291	*sseB*	Unknown
Rv3189	—	Unknown
